# Biofunctional Polyvinyl Alcohol/Xanthan Gum/Gelatin Hydrogel Dressings Loaded with Curcumin: Antibacterial Properties and Cell Viability

**DOI:** 10.3390/gels11100764

**Published:** 2025-09-23

**Authors:** María José Rivera, Alejandro Cament, Manuel Ahumada, Teresa Corrales, Verónica García, Jesús L. Pablos, Javiera Osorio, Giselle Ramos-González, Leslie Vargas-Saturno, Marcelo Ezquer, J. Andrés Ortiz

**Affiliations:** 1 Laboratorio Química de Biomateriales, Departamento de Ciencias del Ambiente, Facultad de Química y Biología, Universidad de Santiago de Chile (USACH), Santiago 9170022, Chile; 2 Escuela de Biotecnología, Facultad de Ciencias, Ingeniería y Tecnología, Universidad Mayor, Camino La Pirámide 5750, Santiago 8580745, Chile; 3 Centro de Nanotecnología Aplicada, Facultad de Ciencias, Ingeniería y Tecnología, Universidad Mayor, Camino La Pirámide 5750, Santiago 8580745, Chile; 4 Grupo Fotoquímica de Polímeros, Instituto de Ciencia y Tecnología de Polímeros (CSIC), Juan de la Cierva 3, 28006 Madrid, Spain; 5 Departamento de Ciencia y Tecnología de los Alimentos, Universidad de Santiago de Chile, Alameda 3363, Estación Central, Santiago 9170022, Chile; 6 Departamento Química en Ciencias Farmacéuticas, U.D Química Inorgánica y Bioinorgánica, Instituto de Investigación Sanitaria Hospital 12 de Octubre i + 12, Universidad Complutense de Madrid, Plaza Ramón y Cajal s/n, 28040 Madrid, Spain; 7 Centro de Medicina Regenerativa, Facultad de Medicina, Clínica Alemana-Universidad del Desarrollo, Santiago 7610658, Chile

**Keywords:** hydrogel, curcumin, antibacterial activity, cytocompatibility, MRSA

## Abstract

This study explores the development of biocompatible hydrogel dressings incorporating curcumin as an alternative antibacterial agent. In this context, hydrogels were prepared using polyvinyl alcohol, xanthan gum, gelatin, and curcumin as a therapeutic component. FTIR spectroscopy confirmed the successful incorporation of curcumin into the hydrogel matrix, while release profiles demonstrated sustained release. Mechanical testing indicated that xanthan gum reduced elongation and strength in hydrogels, while the combination of xanthan gum and gelatin increased stiffness without loss of elasticity. Curcumin had no major effect on the tensile and rheological properties, preserving the structural integrity of the hydrogels. The hydrogels demonstrated antibacterial activity against *Pseudomonas aeruginosa* and *Staphylococcus aureus* ATCC strains, as well as multidrug methicillin-resistant *Staphylococcus aureus* (MRSA) clinical isolates. Biocompatibility was confirmed through viability assays with immortalized human keratinocytes (HaCaT) and adult human dermal fibroblasts (HDFa), showing no acute cytotoxic effects after 48 h of exposure. Their effective action against clinically relevant bacteria and high cytocompatibility position these hydrogels as promising candidates for infection management and antibiotic resistance mitigation in wound care applications.

## 1. Introduction

Tissue engineering has emerged as a key discipline in regenerative medicine, offering innovative solutions to the challenges of modern healthcare. This interdisciplinary field integrates engineering, biology, and materials science to create functional substitutes for damaged tissues and organs, to restore or enhance their function, and ultimately improve patients’ quality of life [1]. A significant advancement in this field is the use of biocompatible materials, such as synthetic polymers and hydrogels, which, in combination with physicochemical stimuli, promote cell growth and tissue regeneration [2].

One of the leading causes of bacterial infections in hospital settings and in the community is methicillin-resistant *Staphylococcus aureus* (MRSA). The clinical manifestations of MRSA range from asymptomatic colonization of the nasal mucosa to severe tissue infections. Among these, skin infections are particularly concerning due to their high mortality rate and potential to become life-threatening if not correctly treated [3]. Although current therapeutic options against MRSA remain limited, there is an urgent need to explore new, safe antibacterial agents capable of effectively targeting this multidrug-resistant pathogen [4].

Conventional wound dressings have shown significant limitations, particularly in the treatment of infections caused by antibiotic-resistant bacteria. While they provide a physical barrier, they do not support effective healing in complex or chronic wounds [5]. In this context, hydrogels have emerged as an effective alternative due to their ability to maintain a moist environment that promotes healing, as well as their biocompatibility, water absorption, biodegradability, and good mechanical strength [5,6,7,8].

A synthetic polymer used to produce hydrogels is polyvinyl alcohol (PVA), a water-soluble material with the mechanical properties required for hydrogel applications. PVA is biocompatible and enhances the structural integrity of hydrogels [9]. However, since PVA-based hydrogels lack sufficient mechanical flexibility and tend to be brittle, PVA is often combined with other polymers to improve their performance for biomedical applications. These combinations form reinforced polymer networks that enhance elasticity, toughness, and functional versatility [10,11]. In addition, the application of freeze–thaw (F/T) cycles to PVA further improves the water absorption capacity and vapor permeability of the hydrogel, while the hydroxyl groups in the constituent polymers facilitate enhanced cell adhesion, making these systems attractive for dressing design [12,13].

Even though numerous studies have reported the use of PVA-based dressings in combination with polysaccharides such as chitosan [14] and alginate [15], xanthan gum (XG) represents an attractive but less frequently explored alternative. Derived from microbial fermentation processes that utilize simple sugars rather than biomass-dependent sources, XG is synthesized by *Xanthomonas campestris*. The resulting polymer is a high–molecular-weight heteropolysaccharide rich in hydroxyl, carboxyl, and other functional groups capable of interacting with PVA [16]. This exopolysaccharide has considerable potential for commercial applications, particularly in medicine, owing to its exceptional matrix-forming capacity, optimal rheological properties, and high biocompatibility. Moreover, XG has been used recently in biomedical applications, such as drug delivery, scaffold creation, and wound healing [17,18].

Gelatin (Gel) is another biopolymer of interest that is well-known for its low antigenicity, biodegradability, and biocompatibility conditions. Derived from collagen, a major component of the extracellular matrix (ECM), its hydrophilic nature makes it ideal for blending with other polymers to improve mechanical performance, facilitating its use in wound care. In addition, Gel mimics ECM proteins, thereby promoting cell adhesion and supporting tissue regeneration [19].

In this study, curcumin (CUR) was selected to develop a dressing with enhanced bioactive properties that is effective against antibiotic-resistant microorganisms such as MRSA. CUR is a natural polyphenolic compound approved by the FDA and possesses various therapeutic properties, including antioxidant, antibacterial, antitumor, anti-inflammatory, and wound-healing effects [20,21,22]. Its effectiveness against common wound pathogens such as *Staphylococcus aureus* positions it as a promising alternative to antibiotics in treating complications associated with infected wounds [23,24]. While CUR-loaded hydrogels have been previously developed [25,26], polymeric multi-network systems specifically based on PVA, XG, and Gel have not yet been reported for the design of biofunctional hydrogels. This distinctive combination provides a novel approach for developing potential biomaterials with antibacterial properties suitable for wound dressing applications.

Specifically, this study aimed to develop novel PVA/XG/Gel/CUR hydrogel dressings with antibacterial and biocompatible properties. The dressings were designed to demonstrate antibacterial activity against multidrug-resistant bacteria commonly associated with skin infections, offering a promising alternative to conventional antibiotics and cytocompatibility. Although PVA-based hydrogels combined with other natural polymers have been widely studied, the specific formulation of these multi-network systems has not been characterized in terms of their mechanical performance, antibacterial efficacy against clinically relevant skin-infecting bacteria, and biocompatibility. In this study, the swelling, thermal, mechanical, rheological, release, antibacterial, and cell viability properties of these novel dressings are reported. We hypothesized that this proposed combination would be capable of reducing antibiotic-resistant bacteria while providing release of a pharmacologically active compound, with potential application as a dermal drug delivery device.

## 2. Results and Discussion

### 2.1. Development and Swelling Assessment of Hydrogel Dressings

The hydrogel dressings were elaborated using the freeze–thaw method, in which crystallization during freezing and the formation of junction points or crystallites upon thawing result in the three-dimensional hydrogel network. In this study, three freeze–thaw cycles followed by lyophilization were conducted to confer porous characteristics to the hydrogel. This process, supported by previous research [27,28], was selected to optimize the swelling ratio while eliminating the need for potentially toxic crosslinking agents meanwhile, the three freeze–thaw cycles allowed for the formation of larger pores, thereby enhancing the hydrogel’s water absorption capacity, which is an essential characteristic for maintaining consistent wound moisture [29]. This structural integrity is further reinforced by non-covalent interactions between the functional groups of the polymers, particularly the hydroxyl and carboxyl groups of XG, the hydroxyl groups of PVA, and the carboxyl, amine, amide, and hydroxyl groups of Gel. In turn, these interactions play a crucial role in the formation of the three-dimensional hydrogel network, considerably contributing to its mechanical stability and durability. During the physical crosslinking process, PVA plays a fundamental role by forming crystalline regions through hydrogen bonding as its polymer chains crystallize during the initial freezing stage. This process progressively increases the crystalline content within the hydrogel, enhancing crosslinking density while simultaneously reducing water absorption capacity, ultimately leading to a lower swelling ratio [30].

**Figure 1:**
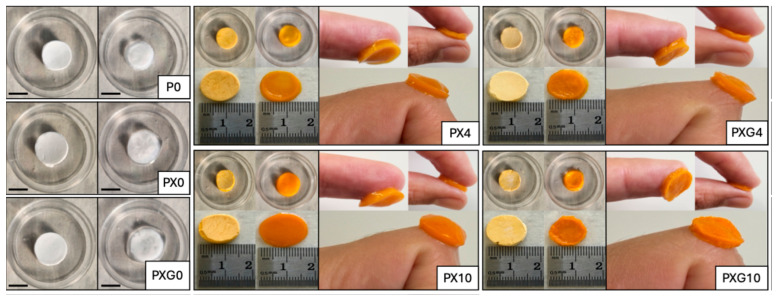
Photographs of lyophilized (**left**) and hydrogel (**right**) dressings based on PVA, xanthan gum, and gelatin with and without curcumin. The dressings were hydrated at pH 5.5 for 72 h. Diameter of samples: 15 mm. Scale bar: 10 mm.

Figure 1 illustrates hydrogel dressings composed of PVA (P0), PVA/XG (PX0), and PVA/XG/Gel (PXG0), both without and with CUR, in their swollen and xerogel forms. It is worth noting that, when PVA was combined with XG and gel, the resulting dressings exhibited greater structural integrity compared to neat PVA. CUR was encapsulated within the hydrogels at 4 wt% and 10 wt%, in accordance with values reported in the literature [31,32,33]. The hydrogels loaded with 4 wt% CUR displayed a slightly orange appearance and demonstrated good CUR dispersion across all formulations.

**Figure 2:**
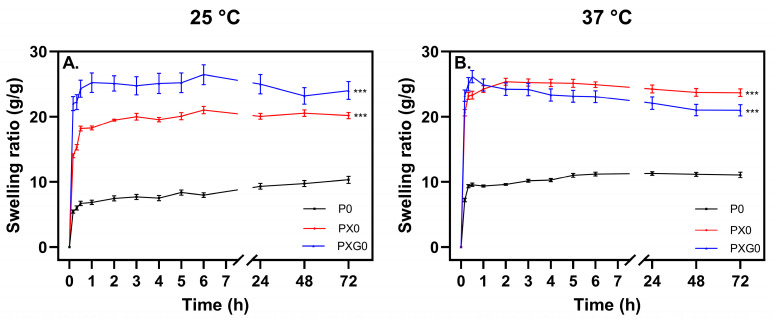
Swelling curves of hydrogel dressings at pH 5.5 (*n* = 6) at (**A**) 25 °C and (**B**) 37 °C. Statistical significance: *** *p* < 0.001 vs. P0.

Figure 2 presents the swelling curves at 25 °C and 37 °C, along with the statistical analysis of hydrogel formulations after 72 h. The results indicate that the hydrogel composed solely of PVA (P0) exhibited the lowest absorption capacity, reaching approximately 6 and 9 times its weight within 30 min at 25 °C and 37 °C, respectively. This behavior is attributed to the formation of a dense crosslinked network due to hydrogen bonding between hydroxyl groups in the PVA matrix.

The PVA/XG (PX) hydrogel exhibited the highest swelling capacity within the first hour among all formulations, absorbing up to 18 times its weight at 25 °C and up to 26 times at 37 °C. These values surpass those reported by Bernal-Chávez et al. [13], who developed hydrogels with PVA (98% hydrolyzed, Mw 31,000–50,000 g/mol, 9.5% *w*/*v*) and XG (0.5% *w*/*v*) through three freeze–thaw cycles.

At 25 °C, the PVA/XG/Gel (PXG) hydrogel exhibited the highest swelling ratio, likely due to the hydrophilic nature of its polymeric components. The interactions between polyanion XG chains, carrying charges in their structure, may have generated repulsive forces that expanded the polymeric matrix. This behavior aligns with the Flory-Rehner theory, which suggests that the presence of free ions influences “ionic” osmotic pressure [34]. Temperature also had a significant effect on swelling. As the temperature increased from 25 °C to 37 °C, higher absorption capacity was observed. This indicates that the polymers enabled faster expansion of the crosslinked network, possibly due to increased polymer chain mobility and the weakening of hydrogen bonds between polymer chains. This effect was particularly evident in the PVA and XG hydrogels, where the absorption index increased significantly at 37 °C compared to 25 °C.

The statistical analysis of swelling indices at 72 h confirmed significant differences among all formulations at both temperatures, with *p* ≤ 0.001. This suggests that each formulation can be independently assessed based on its swelling behavior. However, all formulations except the PVA-only hydrogel exhibited swelling ratios exceeding 15 times their weight for most of the experimental period at both temperatures. It should be noted that maintaining optimal wound moisture is essential for the healing process, as it facilitates cell migration and promotes new tissue formation [35,36]. Additionally, preserving a moist wound environment helps prevent scab formation, which can hinder healing and elevate the risk of infection [37]. The ability of these hydrogels to retain elevated moisture levels further underscores their potential as advanced wound dressings.

### 2.2. Characterization of Hydrogel Dressings

#### 2.2.1. ATR-FTIR Spectroscopic Characterization

**Figure 3:**
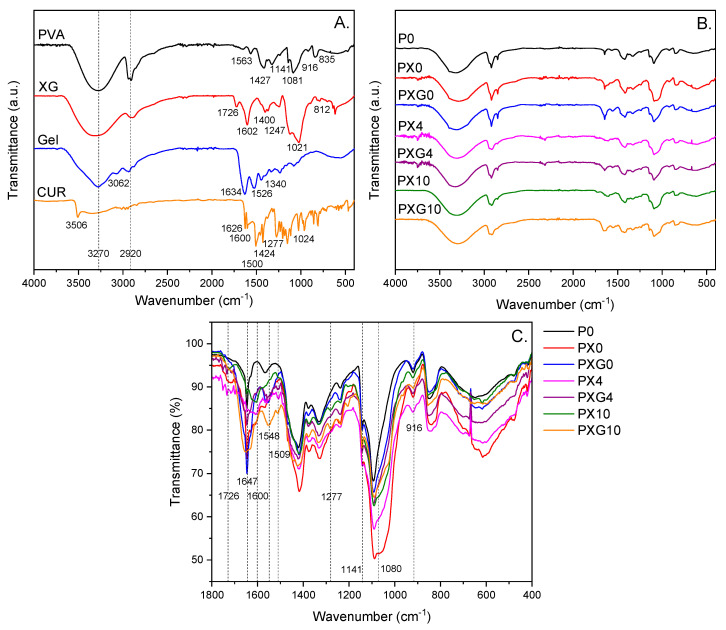
ATR-FTIR spectra in the 4000–400 cm^−1^ region of (**A**) PVA, XG, Gel, and CUR, (**B**) lyophilized dressings without and with CUR (4 wt% and 10 wt%), and (**C**) superposed ATR-FTIR spectra in the 1800−400 cm^−1^ region.

Figure 3 shows the ATR-FTIR spectra of the individual precursors used in the preparation of the hydrogel dressings, including PVA, XG, Gel, and CUR, as well as the ATR-FTIR spectra of the resulting dressings without and loaded with CUR.

The ATR-FTIR spectrum of PVA displays a broad and intense band in the range of 3550–3200 cm^−1^, which can be linked to the ν_s_ of O–H groups from alcoholic groups. The absorption bands between 3000 and 2840 cm^−1^ correspond to C–H stretching vibrations from the hydrocarbon backbone. The bands of lower intensity observed at 1427, 1141, and 1081 cm^−1^ are assigned to methylene δ C–H, ν C–O, and a combination of ν C–O with δ O–H, respectively. In the fingerprint region, weak absorption bands appear at 916 and 835 cm^−1^, which are attributed to C–O and C–C stretching vibrations, respectively [38].

The ATR-FTIR spectrum of XG exhibits characteristic peaks typical of polysaccharides. The broad band around 3330 cm^−1^ corresponds to the ν_s_ of O–H groups, while the peak at 2901 cm^−1^ is attributed to the ν_as_ of C–H bonds in the polysaccharide backbone. The medium-intensity bands at 1726, 1602, and 1400 cm^−1^ are associated with the ν_as_ of C=O in acetyl groups, and the ν_as_ and ν_s_ vibrations of carboxylate (COO^−^) groups, respectively, of pyruvate in β-D-mannopyranosyl and β-D-glucuronic acid residues. The XG spectrum also displays a low-intensity band at 1247 cm^−1^, attributed to ν C–O with δ C–O–H contributions. Two prominent and well-defined bands at 1125 and 1021 cm^−1^ are assigned to the ν_as_ of the C–O–C glycosidic linkage, with overlapping contributions from ν C–O within the pyranosyl ring and δ C–C–O vibrations, respectively. The low-intensity band at 812 cm^−1^ is characteristic of the δ C-H β-glycosidic bond [39,40].

Furthermore, the ATR-FTIR spectrum of Gel displays characteristic vibrational modes associated with the functional groups of a protein. The peaks observed at 3287 and 3062 cm^−1^ are attributed to the ν_s_ N–H and the ν_s_/ν_as_ C–H, respectively. The distinct bands at 1634, 1526, and 1340 cm^−1^ are assigned to the ν_as_ C=O (amide I), δ N–H (amide II), and ν_s_ C=O with contribution of ν C–N (amide III), respectively [41]. The spectrum of pure CUR exhibited characteristic bands at 3512, 2900, and 1626 cm^−1^, corresponding to the stretching vibrations of phenolic O–H groups, ν_s_/ν_as_ of aliphatic and aromatic C–H bonds, and ν C=O with contributions from conjugated aliphatic ν C=C, respectively. Peaks observed between 1600 and 1500 cm^−1^ are attributed to ν C–C vibrations within the aromatic ring. A sharp band at 1424 cm^−1^ is assigned to phenolic ν C–O, while the band at 1277 cm^−1^ corresponds to the enolic C–O group. The absorption at 1024 cm^−1^ is related to the ν_as_ C–O–C. Additionally, bands between 960 and 850 cm^−1^ are assigned to trans-CH bending and C–C skeletal vibrations, respectively [42].

Superposing the ATR-FTIR spectra of the CUR-free formulations (P0, PX0, and PXG0) reveals the characteristic vibrational bands associated with the functional groups of PVA. Additionally, upon incorporation of XG and Gel, a new absorption band is observed at 1726 cm^−1^, along with an increase in the intensity of the band at 1647 cm^−1^; both are attributed to C=O stretching vibrations. A broadening of the signal centered ca. 1080 cm^−1^, associated with the stretching vibrations of the pyranosyl ring, is observed. Furthermore, the incorporation of CUR at 4 wt% and 10 wt% resulted in a proportional increase in the intensity of specific absorption bands. Signals observed at 1600, 1548, 1509, and 1277 cm^−1^—corresponding to phenolic O–H, and aliphatic and aromatic C–C and C–O vibrations characteristic of CUR—confirm its effective incorporation into the polymeric network of all formulations [43]. These spectral findings suggest the successful integration of XG, Gel, and CUR into the PVA-based hydrogel matrix.

#### 2.2.2. Thermal Studies

Table 1 presents the results of thermogravimetric analysis (TGA), which include the 10% mass loss (T_10_), the maximum degradation temperature (T_max_), as well as the differential scanning calorimetry (DSC) results. In the DSC analysis, the glass transition temperature (T_g_) and melting temperature (T_m_) during heating, and the crystallization temperature (T_cc_) during cooling, are identified. Additionally, the enthalpy (ΔH_m_) and crystallinity percentage (X_c_) of the samples are calculated. The TGA shows three degradation zones: the first, close to 100 °C, is associated with water evaporation [44,45,46]; the second, between 200 °C and 340 °C, corresponds to the decomposition of long PVA chains, generating chains of lower molecular weight [47]; and the third, between 400 °C and 600 °C, is related to the decomposition of gelatin [45] and XG [46]. These results indicate that the hydrogels are thermally stable at temperatures above 100 °C, making them suitable for sterilization under high-temperature methods, a critical requirement for biomedical applications [48].

The DSC analysis also confirms the high thermal stability of the samples. The DSC curves showed the three typical physical transitions. The glass transition temperature (T_g_) reflects the state change in the amorphous sections of the hydrogels as the temperature increases, transitioning from a glassy state to a more flexible state, often referred to as “viscous liquid”. However, since the sample has not completely melted at this stage, the temperature ranges in which this transition occurs vary depending on the chemical structure of the hydrogel [48]. An increase in T_g_ was observed compared to PVA, suggesting that polymer interactions affect this temperature, as higher crosslinking or hydrogen bonding can shift T_g_ to higher temperatures [49]. The incorporation of XG into the PVA matrix increases the T_g_ in the system. Adding Gel to the PVA/XG matrix allows the T_g_ to rise, so that the movement of the chains will begin at a higher temperature. This may be due to the crosslinking that exists between the polymers. Meanwhile, the incorporation of 10 wt% CUR made the sample display a higher glassy behavior.

The melting temperature (T_m_) decreased compared to PVA, with a peak associated with XG and gelatin, which is explained by the influence of their pseudoelastic properties. These polymers form bonds during crosslinking and crystallite formation, allowing for a more flexible material with less mobility restriction [13].

The percentage of crystallinity (X_c_) showed that the incorporation of XG into PVA decreases the crystallinity of samples PX0, PX4, and PX10. This effect is likely related to the amorphous nature of XG [13]. In turn, the addition of Gel increases the crystallinity of the PXG0, PXG4, and PXG10 samples, which may be related to the crystalline nature of gelatin [50]. A similar effect was observed in enthalpy: the incorporation of XG decreases enthalpy, while the addition of Gel increases it; however, the enthalpy values of PVA are not reached. Finally, the T_cc_ decreases with the incorporation of XG, probably because the amorphous nature of XG reduces the crystalline domains in the matrix, and the addition of Gel increases T_cc_, which may be due to the crystalline contribution of the latter polymer.

#### 2.2.3. Mechanical Properties

**Figure 4:**
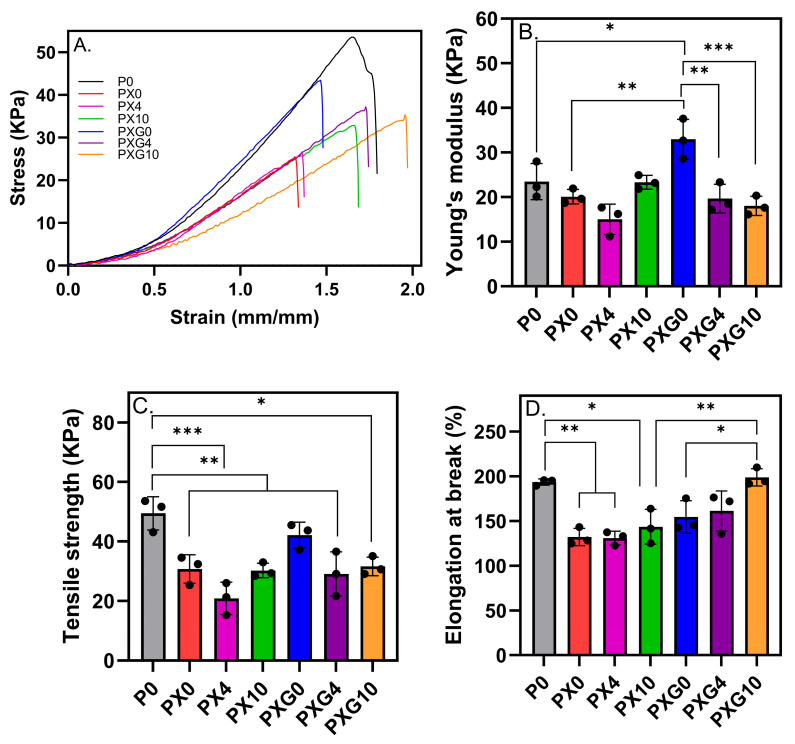
Mechanical properties of hydrogel dressings without and loaded with CUR. (**A**) Stress–strain curves, (**B**) Young’s modulus, (**C**) ultimate tensile strength (UTS), and (**D**) elongation at break (*n* = 3, * = *p* < 0.05, ** = *p* < 0.01, *** = *p* < 0.001).

The mechanical performance of the hydrogel dressings and the influence of CUR incorporation were assessed by measuring stiffness, resistance to applied stress, and deformation capacity prior to fracture. These properties were determined through the calculation of Young’s modulus, ultimate tensile strength (UTS), and elongation at break, as shown in Figure 4.

The control hydrogel P0 exhibited a Young’s modulus of 23.47 ± 4.05 kPa, an elongation at break of 193.47 ± 3.50%, and an ultimate tensile strength (UTS) of 49.43 ± 5.58 kPa. In hydrogels without CUR, the incorporation of XG into the PVA matrix (PX0) did not significantly alter the Young’s modulus but led to a statistically significant reduction (*p* ≤ 0.01) in both UTS (ca. 38%) and elongation at break (ca. 32%), indicating a weakening of the dressing’s deformability. In contrast, the simultaneous addition of XG and Gel into the PVA matrix (PXG0) reinforced the stiffness of the polymeric network (*p* ≤ 0.05) without significantly affecting the strength and flexibility of the material. These findings suggest that while XG disrupts the organization and physical crosslinking of PVA chains—making the network less capable of redistributing mechanical stress—the presence of Gel compensates for the rigidity and fragility caused by XG.

Furthermore, the incorporation of 4 wt% and 10 wt% CUR did not induce statistically significant changes in the tensile properties compared to the CUR-free hydrogel (PX0), indicating that CUR loading did not substantially affect the mechanical performance of the PVA/XG network. In contrast, the addition of 4 wt% and 10 wt% CUR into the PVA/XG/Gel matrix (PXG) resulted in a significant decrease in Young’s modulus of ca. 40%. Regarding elongation at break, 4 wt% CUR did not cause significant changes, whereas 10 wt% CUR led to a significant increase (from 154% to 198%, *p* ≤ 0.01), indicating a concentration-dependent effect on the deformability of the material. It suggests that CUR reduces the stiffness of the PXG system by decreasing the effective crosslinking density and allowing for greater chain mobility.

Overall, the findings indicate that CUR incorporation tends to reduce stiffness and tensile strength, particularly in binary PVA/XG hydrogels. By contrast, the ternary PVA/XG/Gel system (PXG10) preserved flexibility and exhibited a more ductile profile, which may be advantageous for dressing applications that require flexibility and structural integrity under mechanical stress.

#### 2.2.4. Rheological Properties

**Figure 5:**
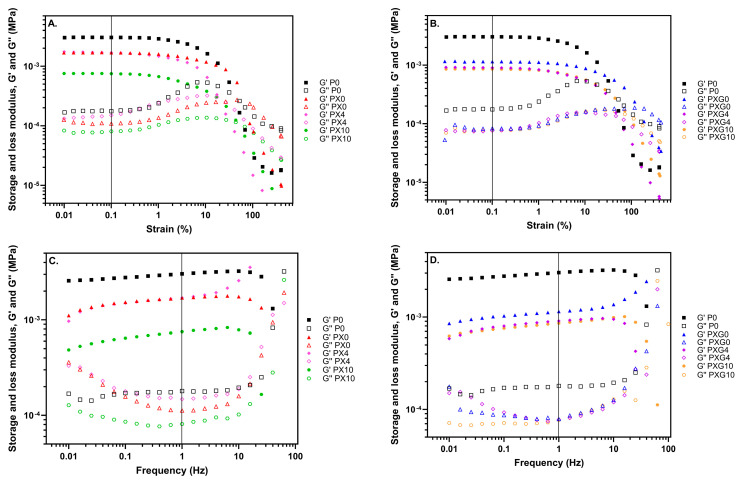
Rheological characterization of hydrogels without and loaded with CUR as a therapeutic agent in terms of storage modulus (G′) and loss modulus (G″). Strain sweep (**A**,**B**), and frequency sweep (**C**,**D**) sequences of hydrogel based on pure PVA, and mixtures of PVA/XG and PVA/XG/Gel with two different loads of CUR.

To evaluate the rheological properties of the hydrogels and analyze the influence of composition on them, rheological studies were conducted. A preliminary dynamic strain sweep test between 0.1% and 400% was conducted to identify the linear viscoelastic range of the hydrogels, that is, the region where the elastic and viscous moduli are strain-independent. As depicted in Figure 5, the storage modulus (G′) remained almost constant within the strain range of 0.01–10%. Furthermore, G′ is higher than the loss modulus (G″) in that interval, which indicates that all hydrogels obtained in this work consistently behave as a viscoelastic solid [51].

Firstly, as seen in Figure 5, the addition of XG causes a decrease in the modulus value (from 3040 Pa to 1690 Pa), which is accentuated slightly more when the mixture of XG and Gel is added to PVA hydrogels (from 3040 Pa to 1150 Pa). In any case, this decrease does not affect the handling properties of hydrogels compared to the hydrogel with pure PVA.

Turning to the inclusion of CUR, this has several effects on the rheological properties of the different hydrogel compositions, resulting in a variation in the values of the storage modulus. Two types of effects are observed: although the addition of XG causes a decrease in the modulus value of the PVA hydrogel, the addition of 4 wt% CUR does not affect the storage modulus value of the hydrogel, while the addition of 10 wt% CUR causes a marked decrease in this value. In contrast, although hydrogels with a mixture of Gel and XG experience a decrease in modulus value compared with pure PVA, this value is not significantly affected by the addition of CUR, even at 10 wt% in the chosen region of viscoelastic behavior. This is a key result that confirms the possibility of introducing a therapeutic agent such as CUR in different proportions without drastically altering its rheological properties. This trend is consistent with the tensile tests, where CUR loading produced minor changes in the mechanical properties of the hydrogels.

**Figure 6:**
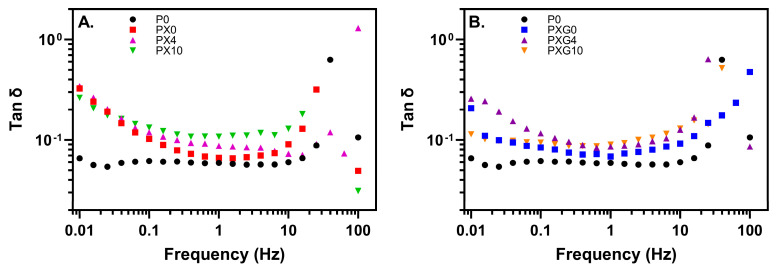
Damping factor (tan δ) as a function of frequency for (**A**) PVA/XG and (**B**) PVA/XG/Gel hydrogels with two different loads of CUR.

Finally, to analyze the elastic balance of different biomaterials, the damping factor (or tan δ) is calculated from the ratio of loss modulus G″ and storage modulus G′ (Figure 6). The damping factor can be used to assess the elasticity of hydrogels. In general, a low tan δ value is related to improved elasticity and is commonly used for comparing the viscoelastic properties of different materials. Values of tan δ < 1 can be attributed to the predominantly elastic behavior of all hydrogels, which is crucial in tissue engineering applications. Although the results show that PVA-based hydrogels have slightly better elasticity and more viscoelastic fluidic nature than other materials containing XG, Gel and CUR and its tan δ value shows less frequency dependence, no evident differences are observed in the elastic behavior of the other materials compared to pure PVA. The values of tan δ (at 1 Hz) range from 0.06 for P0, PX0 and PXG0 to 0.09-0-10 for PX4/PXG4 and PX10/PXG10, respectively, indicating that hydrogels tend to be a solid and stable network with good stiffness [52].

#### 2.2.5. Morphological Analysis

**Figure 7:**
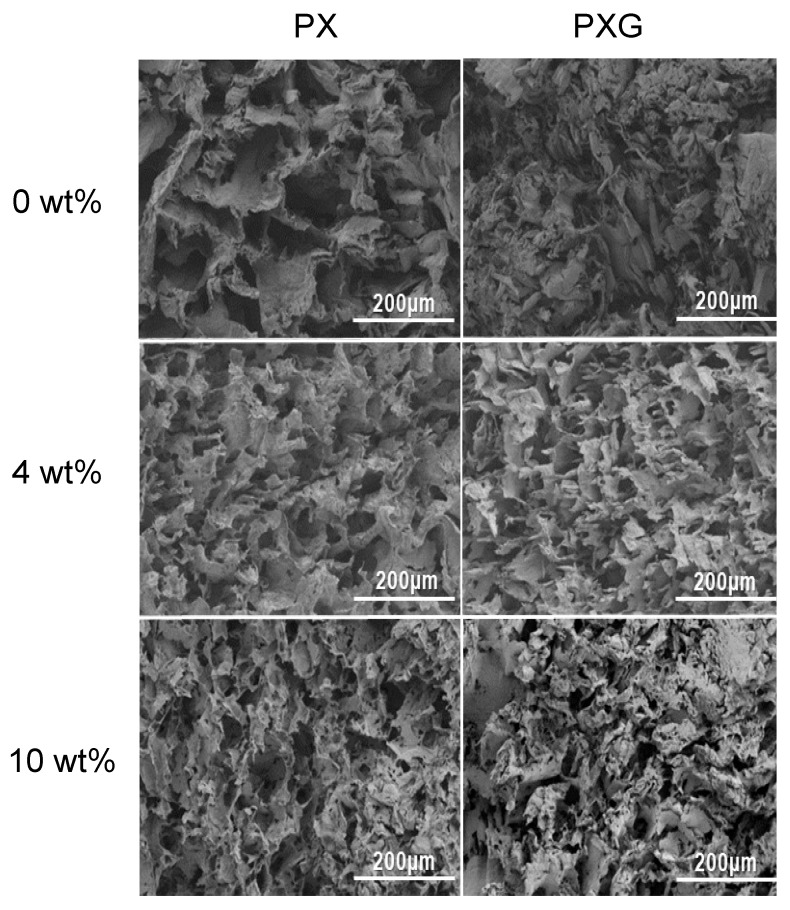
SEM images of the cross-sections of lyophilized PX and PXG dressings containing 0–10 wt% CUR, observed at 200× magnification.

Figure 7 shows the SEM micrographs of lyophilized hydrogel dressings with CUR in concentrations of 0 wt%, 4 wt%, and 10 wt%, displaying a heterogeneous morphology with interconnected pores, regardless of the hydrogel composition. It is observed that the composition of the hydrogel network influences the pore sizes and the thickness of the pore walls, with average pore sizes decreasing after the incorporation of gelatin [53]. After three freeze/thaw (F/T) cycles, the cross-section of PVA/XG/Gel/CUR shows a rough and irregular morphology, which can be explained by the formation of a physically crosslinked network in the lyophilized hydrogel structure due to PVA crystallization. This F/T process increases the porous morphology and promotes the formation of interconnected pores. Porosity makes the hydrogels suitable for use as a matrix for controlled drug release [13], and provides a well-suited space for cell growth, adhesion, proliferation, and migration [54].

Furthermore, this structure increases the permeability of the hydrogels, facilitating the permeation and retention of small molecules, such as water, within the pores. This property promotes the effective absorption and release of water molecules, which increases the elasticity of the hydrogel [55]. Comparisons of PVA/XG/Gel hydrogels with varying CUR contents revealed that higher CUR loading was associated with increased surface roughness. This effect is likely due to the reduced proportion of PVA, XG, and Gel in these formulations, which reduces polymer–polymer interactions and decreases the crosslink density of the matrix [56].

#### 2.2.6. In Vitro Curcumin Release and Analysis of Kinetics Results

**Figure 8:**
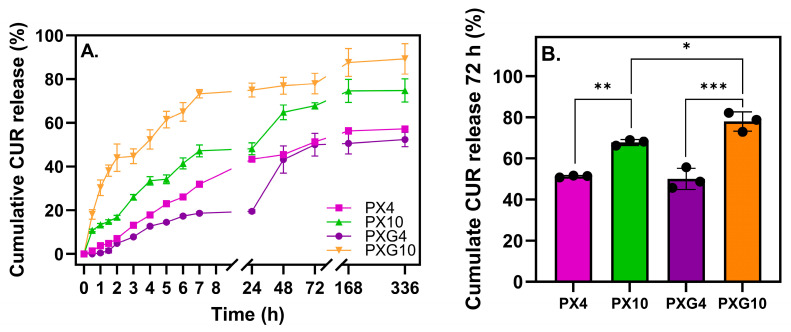
(**A**) Cumulative release curves of CUR at pH 5.5 (*n* = 3) at 37 °C over 2 weeks. (**B**) Statistical analysis of the CUR cumulative release at 72 h. Significant differences were observed with *p* ≤ 0.05 (*), *p* ≤ 0.01 (**), and *p* ≤ 0.001 (***).

In this study, an acetate/ethanol buffer at pH 5.5 (70:30 *v*/*v*) was selected as the release medium, since it provides a physiologically relevant acidic environment that resembles normal skin pH, ensures CUR solubility and stability during the assay, and reflects the preventive role of the hydrogel dressings in protecting wounds under non-infected conditions [32,57,58,59]. Figure 8 shows the release kinetics of CUR from hydrogel dressings containing XG and Gel, loaded with 4 wt% or 10 wt%, over 14 days, along with the statistical analysis of cumulative release at 72 h. The results indicate that all formulations exhibit a gradual and sustained release profile, with a higher initial release within the first 7 h, followed by a slower rate of release in which the cumulative amount tends to remain constant after 48 h. PXG10 displayed the highest cumulative release throughout the study. Within the first 72 h, no significant differences were observed between the formulations containing 4 wt% CUR. In contrast, at 10 wt% CUR, both with and without Gel, a significant increase was observed (*p* ≤ 0.01 and *p* ≤ 0.001, respectively). These findings indicate that higher CUR loading, particularly in combination with Gel, enhances the overall CUR release while maintaining a sustained profile over time.

Table 2 shows the results of different kinetic release models, including zero-order, first-order, Higuchi, and Peppas-Korsmeyer models.

The zero-order model refers to a pharmacokinetic process in which the drug release rate is constant over time, regardless of its concentration. This model did not fit all hydrogels adequately, but the PX4 sample stood out for its linear fit, while the PXG10 sample was the least suited to this model. Meanwhile, the K_1_ values of the first-order model were low and similar among the hydrogels with a 10% CUR concentration. The correlation coefficients were low compared to other mathematical models, reflecting the poor fit of hydrogels to the proposed kinetic model. Therefore, these hydrogels are not characterized by CUR concentration-dependent release [60].

The Higuchi model indicates that drug release is primarily governed by drug diffusion [61]. The results of this model showed large variations in K_H_ depending on the amount of CUR and the combination of polymers. The R^2^ values were very high compared to the other models, with PX10 being the formulation that best fits the Higuchi model. Using the Peppas-Korsmeyer model analysis, the diffusion constant ‘n’ was determined, a parameter that reveals the predominant mechanism governing CUR release. The results obtained indicate that the PXG10 hydrogel exhibits quasi-Fickian diffusion behavior, which is indicated by an ‘n’ value of less than 0.5. This phenomenon is commonly associated with systems characterized by a diffuse release with some limitation. This observation is supported by SEM images, which show smaller pores in PXG10 compared to PXG hydrogels, with average diameters of ca. 22 μm and 45 μm, respectively, suggesting a denser polymer network structure in PXG10 that could hinder CUR release in this system. The PX10 sample had ‘n’ values between 0.5 and 1, indicating anomalous transport through diffusion mechanisms and other mechanisms like polymer chain relaxation [60]. Lastly, the samples with 4% CUR loading exhibited *n* > 1 values, which are typically interpreted as super case II transport, generally associated with polymer matrix erosion. However, the cumulative release curve (Figure 7) shows a plateau in CUR release, suggesting that additional mass transport processes are involved and not erosion alone. Super case II behavior is also characterized by a diffusion rate that is considerably higher than the relaxation rate of the polymer chains constituting the hydrogel [62].

Overall, the cumulative and kinetic release results are consistent with the rheological properties and show a decrease in the G′ modulus as CUR loading increases. The PX4 and PXG4 formulations released a smaller amount of the active compound in a more sustained and controlled manner over time, suggesting a stiffer and denser polymer network, as reflected by their higher G′ values. In contrast, the PXG10 formulation, with a high CUR load, exhibited a lower G modulus compared to the pure PVA matrix (P0), indicating a less densely crosslinked polymer network that facilitates diffusion and results in greater CUR release.

### 2.3. In Vitro Antibacterial Evaluation

**Figure 9:**
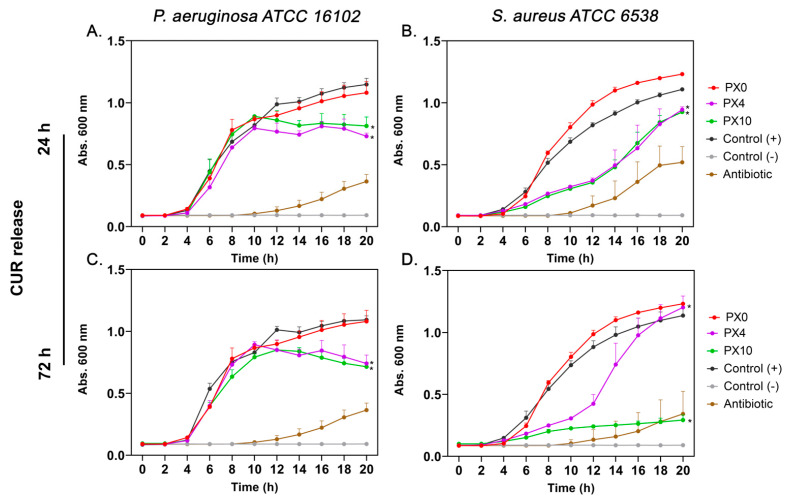
Growth kinetics of *Pseudomonas aeruginosa* ATCC 16102 (**A**,**C**) and *Staphylococcus aureus* ATCC 6538 (**B**,**D**) in the presence of CUR released from PX hydrogels after 24 h (**A**,**B**) and 72 h (**C**,**D**). PX0 (empty hydrogel) did not affect bacterial growth compared to the positive control, while PX4 and PX10 significantly delayed bacterial proliferation, showing a dose-dependent inhibitory effect. The inhibitory effect was more pronounced after 72 h of release. Control (+): bacterial growth without curcumin; Control (–): medium without bacteria; Antibiotic: reference antibiotics used as positive inhibition controls (imipenem 8 μg/mL for *P. aeruginosa*, gentamicin 1 μg/mL for *S. aureus*). Data represent mean ± SD (*n* = 3). Statistical significance: * *p* < 0.0001 vs. Control (+).

The antibacterial activity of CUR released from PX and PXG hydrogels was assessed against *Pseudomonas aeruginosa* ATCC 16102, *Staphylococcus aureus* ATCC 6538, and methicillin-resistant *Staphylococcus aureus* (MRSA) SCL 17064—resistant to cefoxitin, ciprofloxacin, clindamycin, and erythromycin—by monitoring bacterial growth kinetics over 20 h. For PX hydrogels (Figure 9), media containing CUR released for 24 h from PX4 (0.3 µg/mL) and PX10 (0.4 µg/mL) significantly delayed bacterial proliferation in both strains compared to the positive control, with a clear dose-dependent effect. PX0 (empty hydrogel) did not alter bacterial growth. After 72 h of CUR release from PX4 (0.7 µg/mL) and PX10 (1.0 µg/mL), the inhibitory effect became more pronounced, particularly for PX10, which almost completely suppressed bacterial growth during the evaluation period.

**Figure 10:**
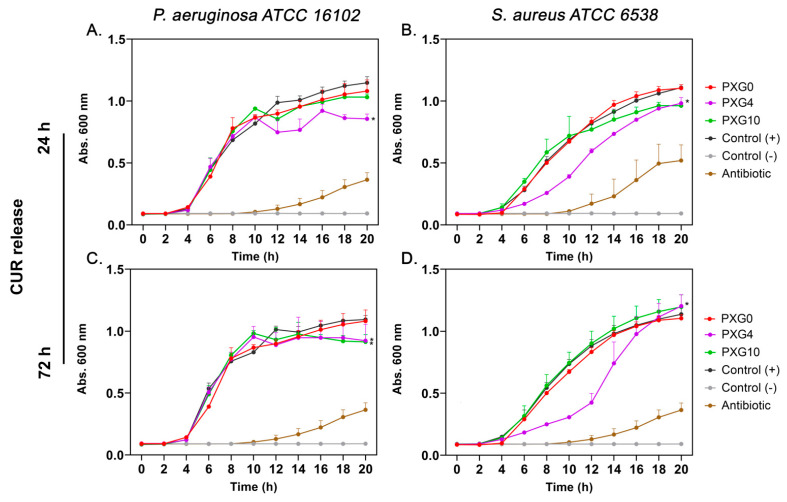
Growth kinetics of *Pseudomonas aeruginosa* ATCC 16102 and *Staphylococcus aureus* ATCC 6538 in the presence of CUR released from PXG hydrogels after 24 h (**A**,**B**) and 72 h (**C**,**D**). PXG0 (empty hydrogel) did not affect bacterial growth compared to the positive control, while PXG4 significantly delayed *S. aureus* proliferation, showing a dose-dependent inhibitory effect. Control (+): bacterial growth without CUR; Control (–): medium without bacteria; Antibiotic: reference antibiotics used as positive inhibition controls (imipenem 8 μg/mL for *P. aeruginosa*, gentamicin 1 μg/mL for *S. aureus*). Data represent mean ± SD (*n* = 3). Statistical significance: * *p* < 0.0001 vs. Control (+).

For PXG hydrogels (Figure 10), no significant inhibitory effect was observed against *P. aeruginosa* at either CUR concentration or release time. However, PXG4 significantly reduced *S. aureus* proliferation after both 24 h and 72 h of release, indicating moderate but measurable antibacterial activity, which increased with longer release times.

**Figure 11:**
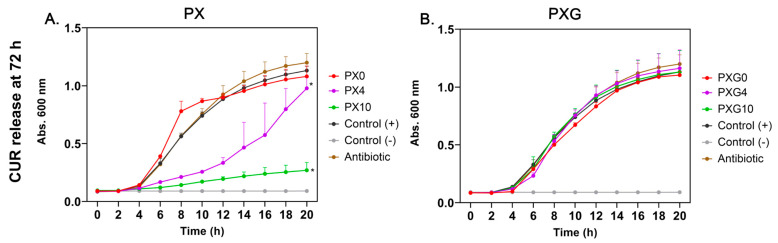
Growth kinetics of MRSA *Staphylococcus aureus* SCL 17064 in the presence of CUR released from PX (**A**) and PXG (**B**) hydrogels after 72 h of incubation. PXG0 and PX0 hydrogels without CUR did not affect bacterial growth compared to the positive control. CUR released from PX4 and PX10 hydrogels significantly inhibited bacterial proliferation in a dose-dependent manner, whereas no significant inhibitory effect was observed for PXG4 or PXG10. Control (+): bacterial growth without CUR; Control (–): medium without bacteria; Antibiotic: ciprofloxacin 2 μg/mL. Data represent mean ± SD (*n* = 3). Statistical significance * *p* < 0.0001 vs. Control (+).

When tested against MRSA (Figure 11), which exhibited resistance when growth kinetics were assessed in the presence of ciprofloxacin (2 μg/mL), CUR released from PX hydrogels (PX4 and PX10) significantly inhibited bacterial growth in a dose-dependent manner. In contrast, PXG hydrogels (PXG4 and PXG10) did not exhibit a statistically significant antibacterial effect. This lack of efficacy can be attributed to the noncovalent interactions—hydrogen bonding and π–alkyl forces—between Gel and CUR, as predicted by molecular docking studies [63]. These interactions suggest that the CUR released from PXG4 and PXG10 at 72 h may undergo partial aggregation in the release medium, either through binding to residual Gel peptides or via self-aggregation, thereby reducing the fraction of free, bioactive CUR and consequently diminishing its antibacterial efficiency. This limitation could be mitigated by optimizing the PVA/Gel ratio, which would reduce Gel–CUR interactions and consequently limit aggregation. Similarly, Musso et al. reported limited or no bacterial reduction at low CUR concentrations in Gel/CUR composite films [64].

The observed variations in the growth kinetics between *P. aeruginosa* (Gram-negative) and *S. aureus* (Gram-positive) could be attributed to differences in cell wall composition. Gram-positive bacteria have a thick peptidoglycan layer that facilitates the interaction and penetration of phenolic hydrophobic molecules such as CUR. In contrast, Gram-negative bacteria possess a more complex cell envelope, with an outer membrane rich in lipopolysaccharides that could act as a barrier, limiting the diffusion of CUR [65].

Overall, these results demonstrate that PX hydrogels exhibit stronger and broader-spectrum antibacterial activity than PXG hydrogels, particularly against Gram-positive bacteria. The effect is enhanced with higher CUR concentrations and longer release periods. Recent in vivo studies have shown that CUR and polysaccharide-based hydrogels can reduce the bacterial burden in infected wounds and promote tissue regeneration, supporting the potential application of the formulations developed in this study as promising biofunctional devices for wound treatment [20,66]. This work also highlights the potential of CUR as a therapeutic option when conventional antibiotics fail to control MRSA infections.

In agreement with these findings, other CUR-based delivery systems have also been explored. For instance, nanocurcumin encapsulated in polyacrylic acid (PAA), polyvinyl alcohol (PVA), and polyethyleneimine (PEI) nanoparticles inhibited the growth of MRSA, with MIC values determined by OD_600_ assays of 0.480, 0.390, and 0.340 mg/mL for PAA, PVA, and PEI nanoparticles, respectively [67]. Similarly, CUR-loaded cyclodextrin-grafted chitosan hydrogels exhibited antibacterial activity against *S. aureus* and *E. coli*, achieving OD_600_ reductions of up to 60% compared to the control [68]. Furthermore, alginate hydrogels formed in situ with CUR host–guest complexes demonstrated high encapsulation efficiency and reduced bacterial colonies by more than 87% in MRSA and *P. aeruginosa* [69].

### 2.4. In Vitro Cell Viability Evaluation

**Figure 12:**
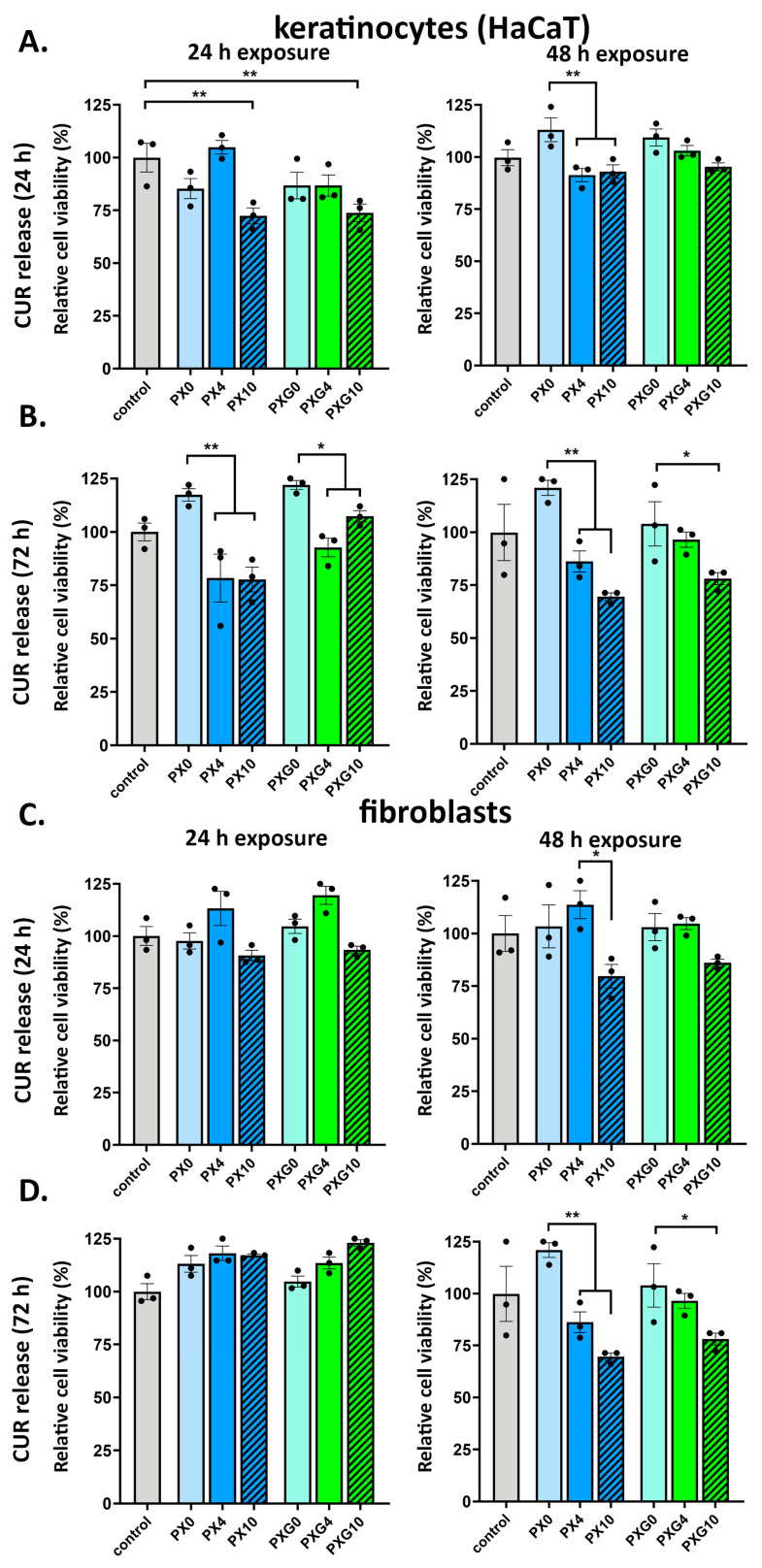
In vitro cell viability of keratinocytes and fibroblasts exposed to curcumin (CUR) released from hydrogel dressings. Panels (**A**,**B**): HaCaT keratinocytes; Panels (**C**,**D**): human dermal fibroblasts (HDFa). Panels (**A**,**C**): cells incubated for 24 h or 48 h with media collected after 24 h of hydrogel release; Panels (**B**,**D**): cells incubated for 24 h or 48 h with media collected after 72 h of release. CUR loadings were 4 or 10 wt% in PX (PVA/XG) and PXG (PVA/XG/Gel) dressings. Cell viability was measured with CellTiter-Blue (ex/em 560/590 nm) and reported as mean ± SEM (*n* = 3). The *y*-axis corresponds to “Relative cell viability (%)”, where the untreated control is cells in serum-free DMEM, defined as 100%. Statistical analysis: two-way ANOVA with Bonferroni post hoc; significance levels: * *p* ≤ 0.05, ** *p* ≤ 0.01.

The biocompatibility of CUR-loaded hydrogels was assessed by analyzing the effect of their released compounds on the viability of keratinocytes (HaCaT) and human dermal fibroblasts (HDFa). Conditioned media were obtained after incubating the hydrogels in a culture medium (DMEM serum free) for 24 and 72 h. Subsequently, the cells were cultured in these media for either 24 or 48 h. As shown in Figure 12, no significant cytotoxic effects were observed under most of the tested conditions, indicating that the hydrogels are generally well tolerated by skin-relevant cell lines.

For keratinocytes (Figure 12A,B and Figure S1), cell viability remained above 80% in nearly all treatment groups. A moderate but statistically significant reduction (~75% viability compared to control) was observed only in cells exposed for 48 h to media containing CUR released after 72 h of hydrogel incubation. This suggests a potential time- and dose-dependent response under prolonged exposure to higher CUR concentrations, although overall cell viability was still acceptable.

In the case of HDFa (Figure 12C,D and Figure S1), exposure to media collected after 24 h of hydrogel incubation resulted in cell viability levels consistently above 90%, regardless of CUR concentration. However, a moderate yet statistically significant reduction (~75% viability) was also noted for fibroblasts incubated for 48 h in a medium containing CUR released over 72 h. This effect was observed only under the most prolonged exposure and highest release condition, suggesting that HDFa has a good tolerance to the hydrogel released components under typical use scenarios.

Overall, HaCaT and HDFa viability remained ≥80% across most treatments, with a moderate decrease to ~75% detected only after 48 h exposure to media obtained after 72 h of CUR release—i.e., under the highest cumulative release condition—supporting a time/dose-dependent but limited effect. Assay controls encompassed serum-free conditions, and exposures were performed with CUR-conditioned serum-free DMEM. Indicating that part of the modest reduction under prolonged exposure may reflect serum deprivation stress and/or protein-binding differences rather than intrinsic hydrogel toxicity. In agreement, cell morphology and confluence were largely preserved (Figure S1), aligning with the quantitative trends in Figure 12. These data support that the dressings are cytocompatible and that the ~75% viability observed in the most stringent in vitro condition is unlikely to be clinically limiting given the expected dilution at the wound bed.

Our results are consistent with previous reports on CUR-incorporated PVA-based polymeric matrices, such as composites with chitosan [70], sodium alginate [71], and silk fibroin [72], which have demonstrated a favorable cytocompatibility profile in the context of wound healing applications. The combination of CUR with hydrophilic polymers appears to mitigate its known low solubility and bioavailability issues while maintaining biocompatibility characteristics.

## 3. Conclusions

Cytocompatible and antibacterial hydrogel dressings made of polyvinyl alcohol, xanthan gum, and gelatin, with curcumin as a bioactive agent, were successfully created. In addition to its well-known antibacterial effects, CUR has poor solubility and low bioavailability in biomedical settings, requiring delivery within a suitable matrix [73]. In this study, hydrogels loaded with different concentrations of CUR showed porous structures and sustained release patterns, controlled by complex mass transport mechanisms involving diffusion, erosion, and polymer chain relaxation. These mechanisms enabled controlled release without burst effects, except in PXG10, improving local CUR effectiveness while minimizing toxicity. Rheological tests confirmed that CUR addition did not significantly affect the viscoelastic properties of the hydrogels, thereby preserving their structural integrity and mechanical performance under stress, consistent with a physical rather than chemical interaction.

Furthermore, the PVA/XG/CUR formulations showed antibacterial activity against ATCC strains *P. aeruginosa* and *S. aureus*, as well as clinical MRSA isolates. Although initial comparisons with standard antibiotics indicated lower effectiveness against model strains, the PX formulations demonstrated better performance against MRSA, supporting the idea that CUR could be a promising agent against drug-resistant bacteria. In this context, future research should investigate dual encapsulation strategies [74], potentially combining CUR with commercial antibiotics to achieve synergistic effects through distinct release kinetics.

While the delivery strategy avoids toxic crosslinkers and shows both cytocompatibility and antibacterial effectiveness, several limitations need to be addressed before moving to clinical use. It is noteworthy that adding Gel did not produce significant improvements compared with formulations without Gel, despite its known pro-regenerative potential [75]. This may be due to the lack of cytotoxicity in the base matrix, which limits gelatin’s observable effects in vitro. Therefore, future research should include in vivo models to evaluate regenerative outcomes such as cell viability, wound closure, and immune response, supported by histological analyses. Encouragingly, all tested formulations showed minimal cytotoxicity, unlike the known toxicity of free CUR [76,77], which emphasizes the importance of vehiculation for targeted delivery. Additionally, albeit not explored here, CUR encapsulation has been reported to improve its stability against photodegradation, temperature changes, and pH fluctuations, which warrants further investigation.

Scalability remains another key consideration. Although the fabrication method aligns with current market practices, challenges such as reproducing porosity and mechanical properties at larger scales, as well as the energy requirements of freeze–thaw cycles, must be addressed to ensure commercial success [78].

In conclusion, CUR was successfully incorporated into PVA/XG/Gel hydrogels, demonstrating controlled release, antibacterial activity against MRSA, and low cytotoxicity to dermal fibroblasts and keratinocytes. Despite current limitations, the proposed freeze–thaw hydrogel system shows promising potential for future wound dressing applications.

## 4. Materials and Methods

### 4.1. Materials

Polyvinyl alcohol (PVA) with an average molecular weight (M_w_) of 85,000–124,000 g/mol and a hydrolysis degree of 99% was obtained from Sigma-Aldrich. Xanthan gum (XG) with a viscometric molecular weight (M_v_) of 1.18 × 10^6^ g/mol and curcumin (CUR) were also supplied by Sigma-Aldrich. Gelatin (Gel, ~60 kDa molecular weight) for microbiology was sourced from Merck. Ethanol P.A. and glacial acetic acid (99% p.a.) prepared the necessary buffer solutions.

### 4.2. Preparation of PVA and Biopolymer-Based Hydrogel Dressings Loaded with Curcumin

The preparation of hydrogel dressings followed the formulations detailed in Table 3. For the P0 formulation, 0.750 g of PVA was mixed with 20 mL of distilled water and transferred to a 50 mL Erlenmeyer flask. PVA was dissolved under continuous stirring at 80 °C for 1.5 h, carefully controlling the temperature to prevent boiling. The PVA solution was then allowed to cool to room temperature.

For the formulations containing XG, Gel, and CUR, the same procedure used for P0 was replicated. Once PVA was fully dissolved, the solutions were cooled to room temperature before incorporating XG, Gel, and CUR. These components were dissolved under gentle stirring at approximately 60 °C. The formulations were subsequently cooled at room temperature, and 0.5 mL of each was transferred into 24-well plates and then frozen at −20 °C for 12 h.

Subsequently, the formulations underwent a crosslinking process consisting of three freeze–thaw cycles, where each cycle included 2 h of freezing followed by 1 h of thawing [13]. Afterward, the formulations were frozen and lyophilized for 18 h. The resulting hydrogels (xerogels) were removed from the plates, forming disc-shaped structures with approximate dimensions of 14 mm in diameter and 0.2 mm in height. Finally, they were stored in a desiccator for further studies.

### 4.3. Characterization

The morphological analysis of the dressings was performed using a scanning electron microscope (SEM) Phillips XL30 MEBA (Hillsboro, OR, USA) with an accelerating voltage of 25 kV. Before imaging, the samples were coated with a thin layer of gold using a Sputter Coater Polaron SC7640 (North Billerica, MA, USA). Functional group identification was done through Attenuated Total Reflectance Fourier-Transform Infrared Spectroscopy (ATR-FTIR) using a PerkinElmer Spectrum Two spectrometer (Waltham, MA, USA). The spectral range covered 4000 to 400 cm^−1^, with 20 scans performed for each sample. The thermal stability of the dressings was assessed using thermogravimetric analysis (TGA) with a TGA analyzer TA-Q500 (New Castle, DE, USA). The measurements were conducted under a nitrogen atmosphere, maintaining a constant gas flow rate of 20 mL/min. Approximately 5 mg of each sample was placed in a 50 μL pan. The samples were subjected to a controlled heating process, starting at 25 °C and increasing to 800 °C at a uniform heating rate of 10 °C/min. Differential scanning calorimetry (DSC) was conducted to evaluate the thermal properties with a PerkinElmer 4000 system (Columbus, OH, USA). Each sample (approximately 5 mg) was sealed in a 40 μL aluminum pan, while an empty pan was used as a reference. The temperature scan ranged from 0 °C to 350 °C at a controlled heating rate of 10 °C/min. All thermal measurements were performed under a nitrogen atmosphere with a constant 2 mL/min flow rate. The melting temperature (T_m_) and melting enthalpy (ΔH_m_) were obtained from the second heating cycle. The percentage of crystallinity (X_c_) was calculated using Equation (1):

(1) $$X_{C}\;=\;\frac{{∆H}_{m}}{{∆H}_{C}}\;\times \;100,$$

where ΔH_m_ represents the sample’s melting enthalpy (J/g), and ΔH_c_ is the enthalpy of fusion for 100% crystalline PVA (138.6 J/g).

### 4.4. Swelling Ratio Determination

The study was conducted using 0.1 M acetate buffer at pH 5.5. Aliquots of 1000 µL were transferred into 6-well culture plates, where the dressings were immersed in the buffer solution and incubated at 25 °C and 37 °C. The mass of the hydrogel dressings was recorded at predefined time intervals over 72 h, and the swelling ratio was then calculated using Equation (2):

(2) $$\mathrm{Swelling}\;\mathrm{ratio}\;=\;\frac{w_{s}\;-{\;w}_{d}}{w_{d}},$$

where w_s_: weight of the swollen hydrogel, wd: weight of the dry dressing.

### 4.5. Study of the In Vitro Release Kinetics of Curcumin

In vitro release assays were conducted in triplicate to evaluate the release profile of the therapeutic compound curcumin (CUR). The experiments were performed in 50 mL Falcon tubes, each filled with 50 mL of acetate/ethanol buffer (pH 5.5, 70:30 *v*/*v*) [32,79] and incubated at 37 °C. The amount of CUR released was determined by extracting 1.0 mL aliquots at specific time intervals over 14 days. After each extraction, 1.0 mL of fresh buffer solution was added to the respective Falcon tube to maintain the total volume. The absorbance of the collected aliquots was measured using a Shimadzu UV-1900i UV-Vis spectrophotometer (Kyoto, Japan) at 430 nm to quantify the released compound.

The release kinetics of CUR were analyzed by applying the mathematical models zero-order, first-order, Higuchi, and Peppas-Korsmeyer, Equations (3)–(6), respectively [80].

(3) $$M_{t}={\;K}_{0}t,$$

where M_t_ is the quantity of CUR released at time t and K_0_ is the zero-order kinetic constant.

(4) $$M_{t}=M_{0}e^{{-K}_{1}t},$$

where M_0_ is the quantity of CUR released at zero time, and K_1_ is the first-order kinetic constant.

(5) $$M_{t}={\;K}_{H}t^{\frac{1}{2}},$$

where K_H_ is the Higuchi kinetic constant.

(6) $$\frac{M_{t}}{M_{\infty }}={\;K}_{p}t^{n},$$

where M_∞_ is the quantity of CUR released at equilibrium. The constants K_p_ and n are characteristic parameters of the solvent-polymer system. The diffusional parameter (n) is influenced by the geometry of the dressing and the physical transport mechanism of the CUR, while the constant k accounts for the polymeric characteristics of the system.

The diffusion constant of CUR into the hydrogels was determined using Equation (7) [60]:

(7) $$\frac{M_{t}}{M_{\infty }}\;=\;4{\left(\frac{\mathrm{Dt}}{\pi l^{2}}\right)}^{\frac{1}{2}},$$

where D represents the diffusion constant of CUR (cm^2^/s) and l corresponds to the thickness of the dry dressing (xerogel) measured by a micrometer.

### 4.6. Mechanical Properties

The mechanical properties of the developed dressings were determined in terms of Young’s modulus, ultimate tensile strength (UTS), and elongation at break, using tensile testing. For each formulation, three rectangular test samples (50 mm × 10 mm × 2 mm; length × width × thickness) were prepared and stored for 24 h prior to testing. The distance between grips was set at 30 mm. Tests were performed with a 50 N load cell at a crosshead speed of 10 mm/min using an Instron EMIC 23-5D (Norwood, MA, USA) at room temperature [81].

### 4.7. Rheological Properties

For assessing the rheological behavior of hydrogels obtained and the influence of adding different components (XG, Gel, and CUR) to the PVA-based hydrogel, rheological tests were conducted using a TA Instruments AR2000 Advanced Rheometer (New Castle, DE, USA) with a 20 mm steel crosshatched plate. To this end, two different kinds of measurements were carried out; first, strain sweep tests, with measurements between 0.1% and 400% strain and a constant frequency of 1 Hz, to determine the linear viscoelastic range. Secondly, frequency sweep tests, with measurements that were carried out in a frequency range between 10^−2^–10^2^ Hz at a specific strain of 0.1% (within the linear viscoelastic range). All tests were performed at 25 °C. Finally, storage and loss modulus (G′ and G″, respectively) were obtained at a frequency of 1 Hz and a strain of 0.1%. In addition, the damping factor (tan δ) was analyzed to study the elastic and viscous behavior of materials.

### 4.8. In Vitro Antibacterial Evaluation

To assess the antimicrobial properties of CUR, it was released into Mueller–Hinton (MH) broth under sterile conditions. Each hydrogel sample was incubated under agitation in 3 mL of MH broth at 37 °C for 24 and 72 h, respectively, in triplicate. To determine the CUR present in the conditioned media, an aliquot was quantified by UV–Vis spectrophotometry at 430 nm. After 24 h hydrogel incubation, CUR concentrations were 0.3 μg/mL (4 wt% loading) and 0.4 μg/mL (10 wt% loading); after 72 h hydrogel incubation, CUR concentrations were 0.7 μg/mL (4 wt% loading) and 1.0 μg/mL (10 wt% loading). The antimicrobial activity of the released CUR was assessed using a broth microdilution assay against *Pseudomonas aeruginosa* ATCC 16102, *Staphylococcus aureus* ATCC 6538, and methicillin-resistant *Staphylococcus aureus* (MRSA) SCL 17064 at a defined concentration. Bacterial cultures in the exponential growth phase were adjusted to a final concentration of 10^5^ CFU/mL. Then, 100 μL of bacterial suspension and 100 μL of CUR released at a concentration of 1/2× were added to each well of a 96-well microplate. The plate was incubated at 37 °C, and the optical density at 600 nm (OD_600_) was measured every 2 h over 20 h to obtain the growth kinetics of each microorganism [82].

In addition to the test samples, appropriate controls were included in each assay: a positive growth control (bacteria without treatment), a positive inhibition control (bacteria in the presence of the corresponding antibiotic, such as imipenem and gentamicin), and a blank (MH broth only, without bacterial inoculum).

### 4.9. In Vitro Cell Viability Evaluation

To assess the effect of CUR on cell viability and proliferation, adult human dermal fibroblasts (C0135 ThermoFisher Scientific, Waltham, MA, USA) and keratinocytes (HaCaT purchased from Cyton, USA) were cultured in the presence of CUR released from hydrogels. The release of CUR was performed under sterile conditions by incubating each hydrogel under agitation in 3 mL of Dulbecco’s Modified Eagle Medium (DMEM) at 37 °C for 24 and 72 h, respectively. To determine the CUR present in the conditioned media used for the viability assays, an aliquot was quantified by UV–Vis spectrophotometry at 430 nm. After 24 h of hydrogel incubation, CUR concentrations were 0.3 μg/mL (4 wt% loading) and 0.4 μg/mL (10 wt% loading); after 72 h of hydrogel incubation, CUR concentrations were 1.0 μg/mL (4 wt% loading) and 0.7 μg/mL (10 wt% loading). Conditioned media were applied to cells at 1/2×, yielding final in-well CUR levels of 0.15 and 0.20 μg/mL (from 24 h media) and 0.50 and 0.35 μg/mL (from 48 h media), respectively.

Cells were seeded into 96-well plates at a density of 5 × 10^3^ cells/well in DMEM supplemented with 10% fetal bovine serum (FBS, HyClone, Marlborough, Australia) and 1.16 mg/mL gentamicin (Sanderson, Laurel, MS, USA). Cells were allowed to adhere overnight at 37 °C in a humidified incubator with 5% CO_2_. Exposures were carried out in DMEM serum-free conditioned with CUR released from the hydrogels; assay controls included serum-free DMEM (untreated control).

After 24 and 48 h of incubation, cell viability was assessed using the CellTiter-Blue Assay (#G8081 Promega, Madison, WI, USA), following the manufacturer’s instructions. Briefly, 20 μL of the reagent was added to each well containing 100 μL of culture medium and incubated for 2 h at 37 °C. Fluorescence was recorded in a Turner BioSystems Modulus fluorometer (Turner BioSystem Inc., Sunnyvale, CA, USA) (ex/em 560 nm/590 nm).

As control for curcumin auto-fluorescence and potential inner-filter effects, for each experimental condition, a matched cell-free blank was prepared for each experimental condition; this consisted of the corresponding conditioned DMEM (containing the released CUR at the same concentration) plus CellTiter-Blue reagent, but without cells. Fluorescence of this matched blank (ex/em 560/590 nm) was subtracted from the signal of the corresponding wells containing cells. Background-corrected values were then normalized to the untreated control (100% viability). A medium-only blank (DMEM + CellTiter-Blue, no cells, no CUR) was also included.

Each condition was tested in triplicate. Controls included an untreated group (cells in DMEM without FBS), a positive group (cells in DMEM plus 10% FBS), and a blank (DMEM, CUR obtained from the hydrogel without cells). Results were expressed as a percentage of viable cells relative to the untreated cells group.

### 4.10. Statistical Analysis

A two-way analysis of variance (ANOVA) was performed to assess statistically significant differences between groups, followed by Bonferroni’s post hoc test for multiple comparisons. Statistical significance was considered at the following levels: *p* ≤ 0.05 (*), *p* ≤ 0.01 (**), and *p* ≤ 0.001 (***).

## Figures and Tables

**Table 1:** Thermal properties of hydrogel formulations determined by TG and DSC analyses.

Sample	TG Analysis		DSC Analysis				
	T_10_ (°C)	T_max_ (°C)	T_g_ (°C)	T_cc_ (°C)	T_m_ (°C)	ΔH_m_ (J/g)	X_c_ (%)
P0	240.2	257.0	75.0	192.2	224.6	47.2	34.0
PX0	234.9	284.2	83.2	152.9	190.8	17.5	12.6
PXG0	245.9	307.7	87.5	173.4	204.8	28.0	20.2
PX4	237.0	285.7	87.9	140.8	192.5	22.7	16.4
PXG4	246.5	299.7	86.0	173.6	207.3	31.7	22.9
PX10	238.0	288.3	85.5	142.8	195.8	21.3	15.4
PXG10	245.4	339.1	89.4	170.0	205.5	29.8	21.5

T_10_: decomposition temperature at 10% weight loss. T_max_: temperature for maximum rate of weight loss. T_g_: glass transition temperature. T_cc_: cold crystallization transition temperature. T_m_: melting transition temperature. X_c_: percentage of crystallinity. Equipment error ± 2 °C.

**Table 2:** Parameter values of mathematical models in the study of CUR release kinetics.

Dressing	Zero Order		First Order		Higuchi		Peppas-Korsmeyer			
	K_0_	R^2^	K_1_	R^2^	K_H_	R^2^	K_p_	n	R^2^	D × 10^4^ (cm^2^ s^−1^)
PX4	4.64	0.971	0.83	0.925	3.35	0.961	0.06	1.20	0.984	3.443
PXG4	2.43	0.946	2.18	0.817	0.27	0.991	7 × 10^−3^	3.33	0.970	0.430
PX10	6.28	0.944	0.27	0.957	14.14	0.999	0.20	0.54	0.901	0.225
PXG10	13.65	0.804	0.25	0.735	38.75	0.995	0.36	0.49	0.936	0.424

K_0_: zero-order kinetic constant. K_1_: first-order kinetic constant. K_H_: Higuchi kinetic constant. K_p_: Peppas-Korsmeyer kinetic constant. n: Peppas-Korsmeyer diffusional parameter. D: diffusion constant.

**Table 3:** Composition and nomenclature of curcumin-loaded hydrogel dressings.

Nomenclature	PVA (wt%)	Xanthan Gum (wt%)	Gelatin (wt%)	Curcumin (wt%)
P0	3.75	0	0	0
PX0	2.50	1.25	0	0
PXG0	2.50	0.62	0.62	0
PX4	2.40	1.20	0	4
PXG4	2.40	0.60	0.60	4
PX10	2.25	1.12	0	10
PXG10	2.25	0.56	0.56	10

## Data Availability

The original contributions presented in this study are included in the article/Supplementary Materials. Further inquiries can be directed to the corresponding author.

## Supplementary Materials

The following supporting information can be downloaded at: https://www.mdpi.com/article/10.3390/gels11100764/s1, Figure S1: Representative phase-contrast images (20×) of keratinocytes (HaCaT cell line) and primary human dermal fibroblasts (HDFa) exposed to CUR released from hydrogel dressings.

## Abbreviations

The following abbreviations are used in this manuscript:

MRSA	Methicillin-resistant *Staphylococcus aureus*
HaCaT	Immortalized human keratinocytes
HDFa	Adult human dermal fibroblasts
PVA	Polyvinyl alcohol
XG	Xanthan gum
Gel	Gelatin
F/T	Freeze/thaw
ECM	Extracellular matrix
CUR	Curcumin
DMEM	Dulbecco’s modified eagle medium
