# Supplementary material for: Biofunctional Polyvinyl Alcohol/Xanthan Gum/Gelatin Hydrogel Dressings Loaded with Curcumin: Antibacterial Properties and Cell Viability
Source: Gels. 2025 Sep 23;11(10):764. doi: 10.3390/gels11100764 (PMC12562834; doi:10.3390/gels11100764)
Supplement: Supplementary file 1 [file gels-11-00764-s001.zip › gels-3854291-supplementary.pdf]

Biofunctional Polyvinyl Alcohol/Xanthan Gum/Gelatin Hydrogel Dressings Loaded with Curcumin: Antibacterial Properties and Cell Viability

María José Rivera, Alejandro Cament, Manuel Ahumada, Teresa Corrales, Verónica García, Jesús L. Pablos, Javiera Osorio, Giselle Ramos-González, Leslie Vargas-Saturno, Marcelo Ezquer and J. Andrés Ortiz*.

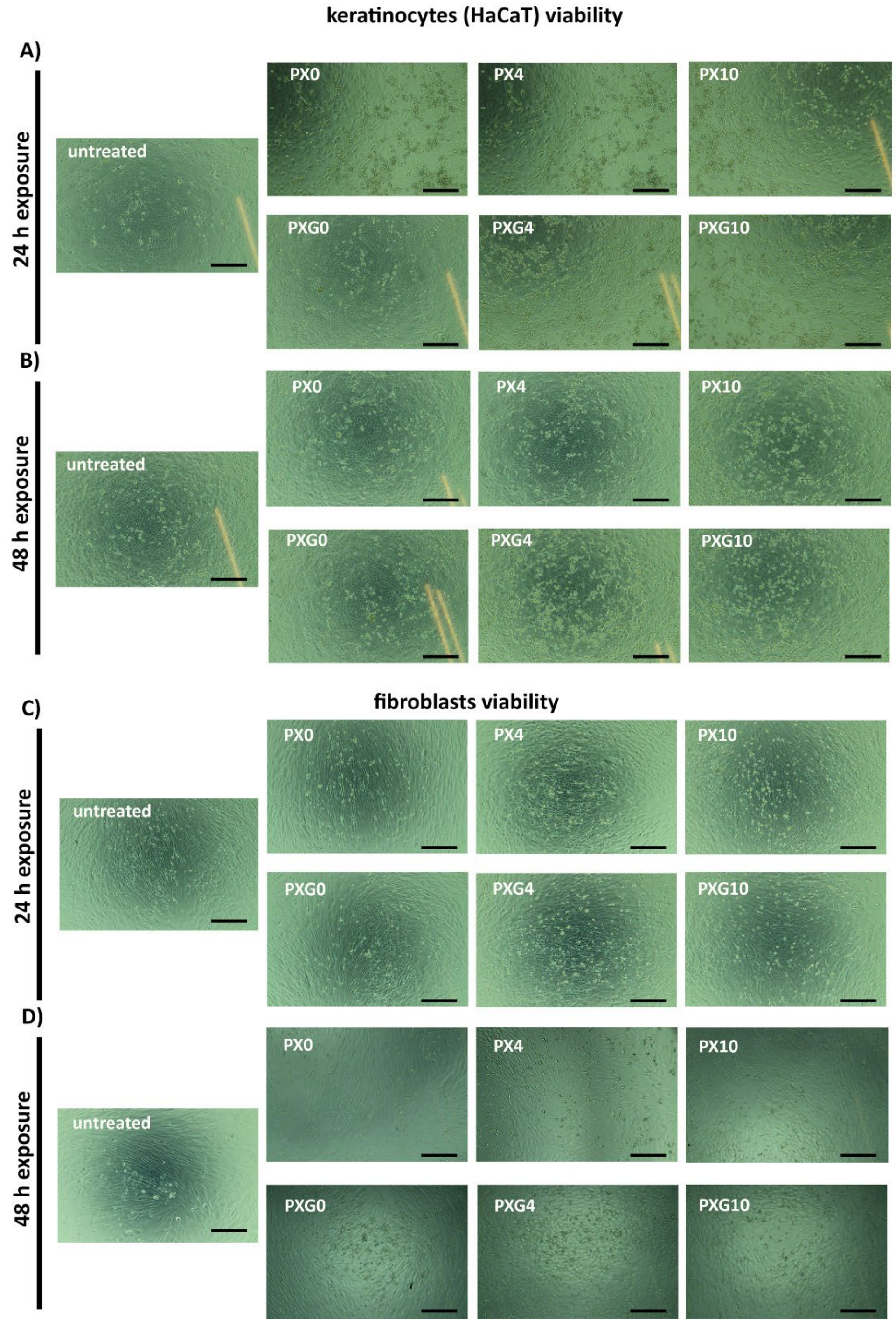

Fig. S1. Representative phase-contrast images (20×) of keratinocytes (HaCaT cell line) and primary human dermal fibroblasts (HDFa) exposed to CUR released from hydrogel dressings.

To evaluate the biocompatibility of the CUR-loaded hydrogel formulations, conditioned media were prepared by incubating PX and PXG hydrogels in complete culture medium at 37 °C for 24 h and 72 h. The resulting media, containing curcumin released from the hydrogels, were then used to culture HaCaT keratinocytes and primary human dermal fibroblast (HDFa) for 24 h or 48 h.

Panels A–B: HaCaT after 24 h (A) and 48 h (B) of exposure; Panels C–D: HDFa after 24 h (C) and 48 h (D) of exposure. Within each panel, images are labeled as PX4, PX10, PXG4, PXG10 for media collected after 24 h and 72 h of hydrogel incubation. Definitions: PX = PVA/XG; PXG = PVA/XG/Gel; numerals denote CUR loading (wt%) in the dressing. Controls: PX0 and PXG0 (no CUR) and untreated (complete culture medium without hydrogel exposure). Scale bar: 200 μm. The images illustrate preserved morphology and confluence for most conditions, in line with quantitative viability data in Fig.12.
